# Advancing Rare-Earth Separation by Machine Learning

**DOI:** 10.1021/jacsau.2c00122

**Published:** 2022-06-15

**Authors:** Tongyu Liu, Katherine R. Johnson, Santa Jansone-Popova, De-en Jiang

**Affiliations:** † Department of Chemistry, University of California, Riverside, California 92521, United States; ‡ Chemical Sciences Division, Oak Ridge National Laboratory, Oak Ridge, Tennessee 37831, United States

**Keywords:** critical materials, rare-earth elements, machine learning, solvent extraction, ligand design, lanthanide separations

## Abstract

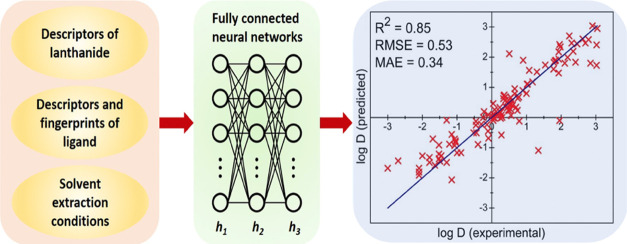

Constituting the bulk of rare-earth elements, lanthanides need to be separated to fully realize their potential as critical materials in many important technologies. The discovery of new ligands for improving rare-earth separations by solvent extraction, the most practical rare-earth separation process, is still largely based on trial and error, a low-throughput and inefficient approach. A predictive model that allows high-throughput screening of ligands is needed to identify suitable ligands to achieve enhanced separation performance. Here, we show that deep neural networks, trained on the available experimental data, can be used to predict accurate distribution coefficients for solvent extraction of lanthanide ions, thereby opening the door to high-throughput screening of ligands for rare-earth separations. One innovative approach that we employed is a combined representation of ligands with both molecular physicochemical descriptors and atomic extended-connectivity fingerprints, which greatly boosts the accuracy of the trained model. More importantly, we synthesized four new ligands and found that the predicted distribution coefficients from our trained machine-learning model match well with the measured values. Therefore, our machine-learning approach paves the way for accelerating the discovery of new ligands for rare-earth separations.

## Introduction

Rare-earth elements (REEs), including the 14 lanthanides, yttrium, and scandium, are recognized as critical materials vital to many technologies.^1−4^ Due to their similar properties, REEs are difficult to separate from one another.^5^ Solvent extraction is the most extensively used process to separate lanthanides on an industrial scale. This process employs an organic ligand (extractant or complexing agent) in a nonpolar, water-immiscible organic solvent (org) to extract trivalent lanthanides, Ln(III), from an aqueous (aq) solution. The extraction performance is expressed as a distribution ratio for each Ln(III), *D =* [M^3+^]_org_/[M^3+^]_aq_. High *D* values indicate better extraction efficiency and imply the formation of stable Ln(III) complexes in the organic phase. Ligands that show great promise in REE separations include diglycolamides (DGA),^6−9^ alkylated bis-triazinyl pyridines (BTP),^10^ and 2,9-bis-lactam-1,10-phenanthroline (BLPhen),^11,12^ among others.^13−15^ Extraction performance is also impacted by experimental conditions, including solvent, temperature, and volume of each phase. Organic solvents such as toluene,^6^ n-dodecane,^16^ 1-octanol,^17^ and dichloroethane^18^ are commonly used to carry out the liquid–liquid separations.

Innovation in ligand design and discovery is key to achieving more efficient separation of Ln(III)s. Knowledge-based design, followed by the synthesis of new ligands, tends to be low throughput and often relies on trial and error to determine optimized extraction conditions. In addition, quantum chemical calculations of the ligand–metal binding are limited by the solvation model and lack solvation dynamics; usually, the relative change in free energy in reference to a common ligand^19,20^ is predicted instead of directly predicting *D* values for Ln(III) for a specific ligand. These calculations also have limited throughput due to high computational cost.

The data-driven machine-learning (ML) approach allows high-throughput screening of much larger chemical space, and the model will continuously improve as more data are generated. This approach has been increasingly used in predicting important equilibrium properties such as solubility,^21,22^ binding affinity,^23^ p*K*_a_,^24^ adsorption capacities,^25,26^ and partition coefficients of molecules.^27,28^ Hence, there is an opportunity to accelerate the discovery of new ligands for Ln(III) separation using the data-driven ML approach.

Herein, we have developed a predictive model that accurately predicts *D* values for a given ligand by training deep neural nets on experimental data of measured *D* values and by sufficiently representing ligands, Ln(III) ions, and experimental conditions. The model is then tested on four new ligands synthesized, and the predicted *D* values are in very good agreement with the experiment, highlighting its predictive power to enable further high-throughput screening.

## Results and Discussion

### Data and Machine-Learning Workflow

**Figure 1:**
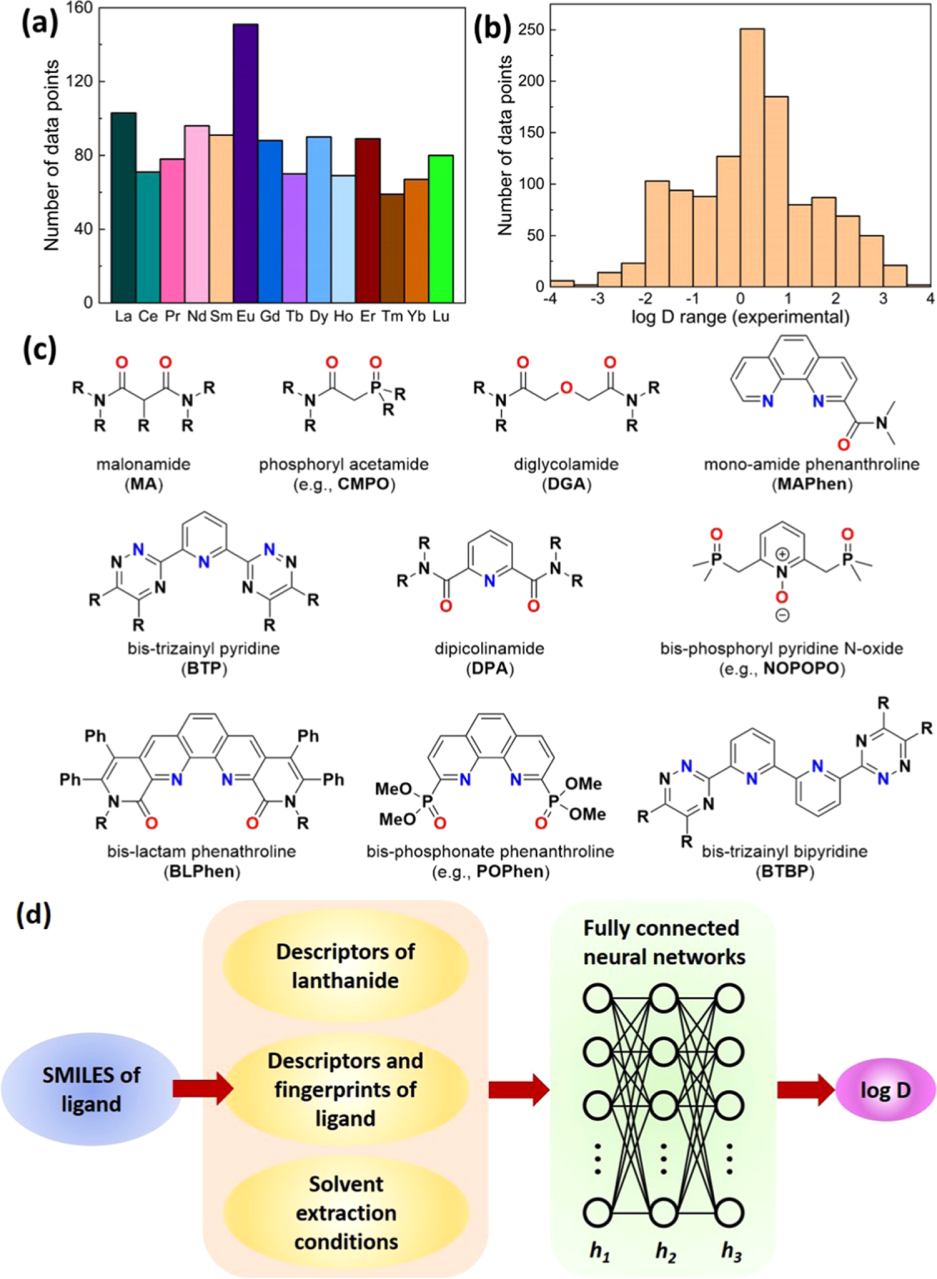
Distribution of the total data set of 1202 experimental log *D* values: (a) based on Ln(III), excluding radioactive Pm(III); (b) the value range. (c) Chemical structures of some representative ligands in the data set. (d) Workflow of predicting log *D* of Ln(III) extracted by a ligand via fully connected neural networks.

In total, 1202 reported *D* values using 109 different ligands were collected from the literature and used to build the data set. Each Ln(III) has more than 60 entries (Figure 1a). The experimental *D* values span eight orders of magnitude: as shown in Figure 1b, log *D* ranges from −4 to +4. Many classes of ligands, including phosphine oxides, amides, and *N*-heterocyclic derivatives, were selected (Figure 1c).^29,30^ 117 data points out of 1202 for 14 Ln(III)s were randomly selected as the validation set.

The workflow of our ML approach is summarized in Figure 1d. The goal or the output is to predict log *D* values, given the input of the specific Ln(III) ion, ligand, and extraction conditions. From the input to the output, there are two major steps: the first step represents Ln(III) ion, ligand, and extraction conditions with descriptors and the second step connects the descriptors to output (log *D*) via neural networks of multiple layers. Below, we first describe the input data in detail and then the training process.

The input data comprise three parts: Ln(III), ligand, and solvent-extraction conditions. Fourteen descriptors are used for each Ln element; see the list in the Supporting Information (SI). The ligand, represented by a string-based name (simplified molecular-input line-entry system or SMILES), is fed into RDKit^31^—a cheminformatics toolkit that automatically generates 208 molecular physicochemical descriptors for the ligand. The RDKit descriptors are then combined with the extended-connectivity fingerprints (ECFPs)^32^ for a more detailed representation of the ligand. Solvent-extraction conditions such as temperature, concentration of the ligand, and physical properties of organic solvents are also part of the input (see the list in the SI). In total, 2291 inputs are used for each output log *D* value; the total data set including the experimental sources of log *D* values is provided in the SI as a separate data file.

### Training and Model Performance

**Figure 2:**
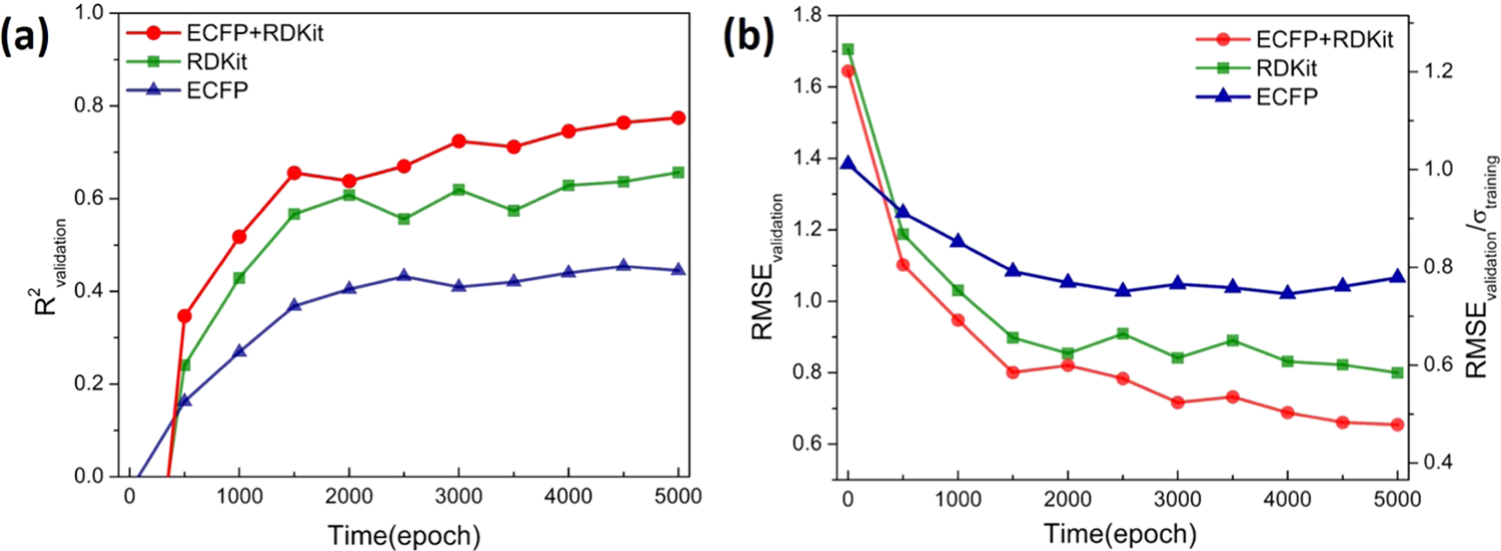
Comparing the three different approaches, RDKit, ECFP, or ECFP + RDKit, to represent ligands, based on the validation set performances of the trained FCNN for predicting log *D* against the experiment in the first 5000 epochs: (a) coefficient of determination, *R*^2^, between the predicted log *D* and experimental log *D* values; (b) root-mean-square error, RMSE, between the predicted log *D* and experimental log *D* values (also measured against the standard deviation, σ, of experimental log *D* values of the training set, right axis). FCNN hyperparameters: 0.00001 learning rate, PReLU activation functions, 0.01 weight decay, three hidden layers, and the number of neurons on each layer = 512, 128, and 16.

Fully connected neural networks (FCNNs) in which every neuron in one layer is connected to every neuron in the next layer were used as the core of our approach for deep learning.^33^ The training of the FCNNs was performed with the PyTorch package.^34^ In each epoch, 80% of the 1085 data points were randomly selected for training. As shown in Figure 2, the coefficient of determination, *R*^2^, between the predicted log *D* and experimental log *D* values of the validation set by using the combination of ECFP and RDKit for the ligands reached a higher value (∼0.80) than that using only ECFP (∼0.45) or RDKit (∼0.65) after 5000 epochs of training (Figure 2a). Likewise, the root-mean-square error (RMSE) of the validation set for the ECFP + RDKit representation decreased more rapidly and achieved a lower value after 5000 epochs (Figure 2b). Hence, the ECFP + RDKit representation of the ligand was used for the subsequent training.

Screenings of hyperparameters are listed in Table S1 from the evaluations of their performances on the validation set. After 5000 epochs, three-hidden-layer models showed better predictions than one or two layers; likewise, the 0.00001 learning rate (i.e., step size in the gradient descent algorithm) was better than 0.001 and 0.000001. On the other hand, different activation functions did not show great differences after 5000 epochs; the activation function introduces nonlinearity when passing inputs from one layer of neurons to the next, mimicking the firing of a neutron for a given input. The most popular activation function is ReLU (rectified linear unit): when passing the ReLU function, the output equals to input when it is positive and zero otherwise. PReLU or parametric rectified linear unit has the same output as ReLU for a positive input but a slightly different output (*y* = 0.25*x*) for a negative input (*x*), instead of 0. We found that the highest *R*^2^ (0.85) for the validation set was reached by the PReLU activation function after 15,000 epochs, with 0.00001 learning rate, 0.01 weight decay, three hidden layers, and the number of neurons on each layer as 512, 128, and 16 (highlighted in bold in Table S1).

**Figure 3:**
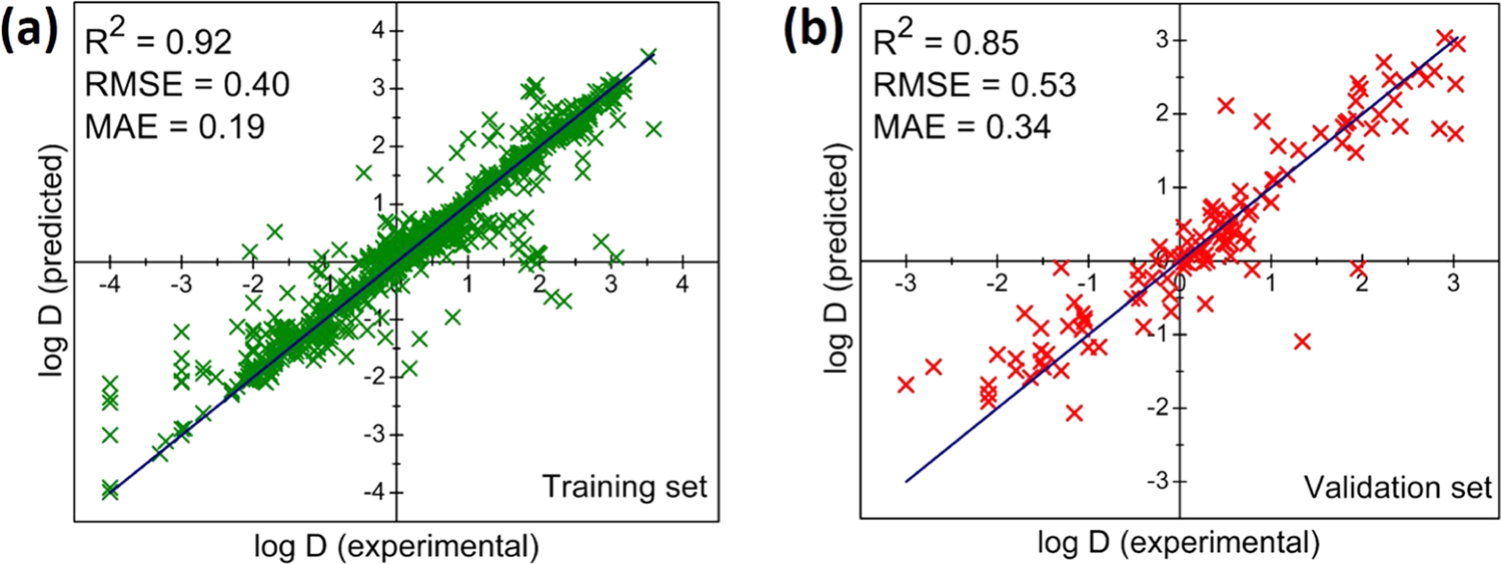
Performance of the best FCNN model. The parity plot between the predicted and experimental log *D* values: (a) training set and (b) validation set.

The best FCNN model’s performance is further shown in Figure 3 as the parity plot. For the 1085 data points used for training, the *R*^2^ value reached 0.92 (Figure 3a) with RMSE of 0.40 and MAE of 0.19. More importantly, the model shows very good performance for the validation set: *R*^2^ = 0.85, RMSE = 0.53, and MAE = 0.34. In other words, this trained model can predict log *D* values with an uncertainty of ∼0.5. Of note, there are some cases with large errors in predicted log *D* values (Figure 3b), and we found that they are mainly from ligands with rare groups (such as -SR) for which we do not have a lot of data in the training set.

### Prediction on New Ligands

**Figure 4:**
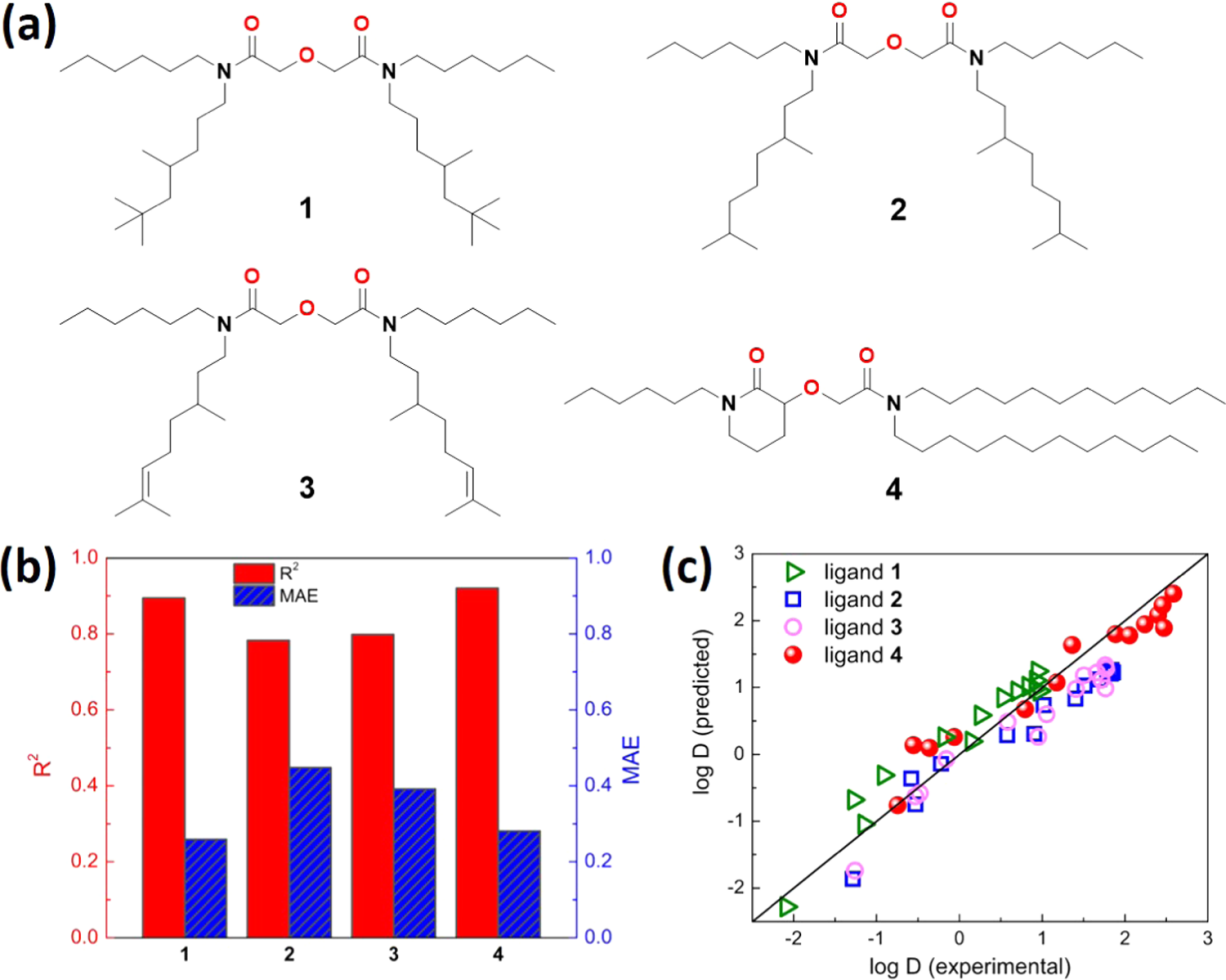
Predictions on new ligands. (a) Chemical structures of new ligands **1**–**4** synthesized for Ln(III) extractions. (b) *R*^2^ and MAE values of predicted log *D* for new ligands **1**–**4** in comparison with the measured values. (c) Parity plots between the predicted and experimental log *D* for ligands **1**–**4**; there are 14 data points for each ligand, representing 14 Ln(III)s extracted at the same conditions.

To further test our FCNN model, four new DGA ligands (**1**–**4** in Figure 4a) with different *N*-alkyl substituents were synthesized in this work (see the SI for details), which are not included in our training or validation set. It is known that subtle changes to the size of *N*,*N*′-alkyl groups affect DGA performance in Ln(III) separation.^8^ The performance of DGAs that incorporate *N*,*N*′-alkyl substituents with branching is rather underexplored, for example, the substituents at γ (e.g., **2** and **3**) and δ (e.g., **1**) positions as opposed to α^35^ and β^36^ positions with respect to the amide nitrogen. Additionally, the introduction of structure-rigidifying elements in DGA, such as the δ-lactam motif in ligand **4**, opens new possibilities for chemically modifying the diglycolamide backbone to further alter separation behavior. The benefits of implementing such structural modifications in DGAs are twofold: (1) extraction strength of Ln(III) can be tuned by varying the steric hindrance around the tridentate binding site and (2) the formation of the third phase in the liquid–liquid setting is more likely to be avoided due to improved hydrodynamic properties of these ligands and their Ln(III) complexes in the nonpolar solvent.

After their successful syntheses, ligands **1**–4 were dissolved in an organic phase and contacted with mixed Ln(III) aqueous solutions in either hydrochloric or nitric acid (see the SI for details). After phase separation, their *D* values were experimentally determined by measuring the aqueous concentration of Ln(III) before and after extraction using inductively coupled plasma optical emission spectroscopy (see the SI for details). To test the accuracy of our ML model to predict log *D* values, we fed these four new ligands together with their separation conditions into our well-trained FCNN model. As shown in Figure 4b, the predicted log *D* values are in good agreement with the experimental values, with *R*^2^ ranging from 0.78 to 0.92; the MAE between the model predictions and experimental observations of log *D* in ligands 1–4 are 0.21, 0.41, 0.38, and 0.22, respectively. Even though this is a small test data set, the observed errors are similar to the validation set MAE of 0.34. This performance is consistent with the validation set shown in Figure 3b. The parity plot of the predicted vs experimental log *D* values for ligands **1**–**4** in Figure 4c highlights the very good performance of this ML model.

Our model can be further improved by incorporating more data into the training data set as they become available, especially for new ligand systems that are not represented in this work. This will help increase the accuracy (*R*^2^) and lower the uncertainty (MAE) of the predicted log *D* values. More importantly, the trained model will allow us to rapidly evaluate new ligands for Ln(III) separation. Recent advances in the automatic generation of molecular structures based on string-based representations^37,38^ provide opportunities to create a large ligand database that can be fed into our ML model for high-throughput screening of new ligands for REE separations. In addition, our approach can be potentially extended to biomolecule-based ligands^39^ and biogenic materials.^40^

In principle, our approach can also be used to screen extraction conditions. There are, however, some practical difficulties, with the main one being that researchers tend to report good extraction conditions while the less desirable conditions were not reported. As a result, the reported extraction conditions usually show limited coverage of the parameter space and there is insufficient data coverage in the extraction conditions in our data set. We think that high-throughput and automated experimentation of extraction conditions would alleviate this insufficiency and make the future effort of predicting optimal extraction conditions with ML highly worthwhile.

## Conclusions

To advance the solvent-extraction separation of rare-earth elements, we have trained deep neural networks on the available experimental data of distribution coefficients measured for hundreds of ligands for 14 Ln(III) ions to accurately and quickly predict their distribution coefficients for a given ligand and the extraction conditions. To best represent the ligands, we found that a combination of molecular physicochemical descriptors and atomic extended-connectivity fingerprints yields the highest accuracy of the trained model on the validation set. We have further explored many combinations of hyperparameters that led to a set of optimal hyperparameters. The best trained model performed well on the validation set: *R*^2^ = 0.85 and RMSE = 0.53. To further test our model, we synthesized four new ligands by modifying the diglycolamide (DGA) backbone and side chains and measured their log *D* values for Ln(III) ions; we found that the predicted distribution coefficients from our trained neural network agree well with the measured values. One can envision that our neural network can now be used to quickly predict log *D* values of Ln(III) ions for thousands to hundreds of thousands of ligands once they are generated. These log *D* values can be further evaluated to screen ligands for separation factors, that is, the ratios of log *D* values. Therefore, this work paves the way for further high-throughput screening of ligands to accelerate the discovery of new ligands for REE separations.

## Methods

### Data Collection

All 1202 log *D* values of lanthanide extraction in our database were collected from the scientific literature, where a single neutral ligand was the only extractant used to extract Ln(III) from the aqueous phase to the organic phase consisting of one or two different solvents. The complete input data and log *D* values of the training and validation sets, as well as those of the new ligands **1**–**4** synthesized in this work, are provided in a separate Excel file as additional Supporting Information. For each data point (one row entry in the Excel file), the inputs (columns) include sequentially the representation of the ligand, descriptors of the extraction conditions, and descriptors of the lanthanide. The source reference of each extraction data point is labeled in the last column in the training and validation sets (but not used for deep learning).

### Representation of Ligands

The first 2,048 inputs of each data point are extended-connectivity fingerprints^32^ (ECFP) of the ligand; the next 208 inputs are RDKit descriptors.^31^ They are both generated from the simplified molecular-input line-entry system (SMILES) expression of the ligand by the DeepChem package.^41^ Chirality is considered in ECFP, and other parameters use default settings: radius of fingerprint = 2, length of the generated bit vector = 2,048, bond order considered, and feature descriptors not used. RDKit descriptors use default parameters: binary descriptors of fragments like “fr_XXX” are returned and avg = True for the IPC (information of polynomial coefficients) descriptor^42^ to return the information content divided by the total population. The names of the 208 descriptors returned by the RDKit module are listed in the Excel file, including molecular weight, number of valence electrons, partial charges, electrotopological state indexes, etc.

### Descriptors of the Extraction Conditions and Lanthanides

Following the ligand’s ECFP and RDKit data in inputs (columns) of the Excel file are the descriptors of the extraction conditions and the extracted lanthanide. The detailed lists are provided in the SI. Descriptors of the Lanthanides.

### Details of the Deep Learning Model and the Training Process

The training of fully connected neural networks (FCNNs) is performed via the PyTorch package (version 1.9.1)^34^ with L1 type loss function, SGD optimizer, and L2 regularization for weight decay. The weight initializations obey the default normal distributions. Mean-absolute error (MAE), root-mean-square error (RMSE), and coefficient of determination (*R*^2^) as calculated via the scikit-learn module were used as metrics for evaluation during the training process.^43^

### Synthesis of Ligands 1–4 and Solvent-Extraction Experiment

The syntheses and characterization of ligands **1**–**4** are described in detail in the SI. For extraction of Ln(III) with **1**–**3**, a 750 microliter (μL) aqueous phase containing 7 mM Ln(III) (0.5 mM of each Ln(III)) in 3 M HCl was contacted with an equal volume of preequilibrated organic phase containing 0.1 M of the desired DGA (**1**–**3**) in 30% v/v Exxal 13/Isopar L. The two phases were contacted using a 1:1 ratio of organic/aqueous solution volume by end-over-end rotation in individual 1.8 mL capacity snap-top Eppendorf tubes using a rotating wheel in an airbox set at 25.5 ± 0.5 °C. Contacts were performed in triplicate with a contact time of 1 h. The samples were centrifuged at 1811*g* for 2 min at room temperature to separate the phases. Each triplicate was then subsampled using a 500 μL aliquot of the aqueous phase transferred to individual polypropylene tubes and diluted with 4% HNO_3_ for analysis. Two samples of the initial lanthanide solution were similarly prepared. The area under each observed emission peak in inductively coupled plasma optical emission spectroscopy was used for determining the concentration of Ln(III) in each solution. For extraction of Ln(III) with **4**, a 500 microliter (μL) aqueous phase containing 7 mM Ln(III) (0.5 mM of each Ln(III)) in 1 M HNO_3_ was contacted with an equal volume of preequilibrated organic phase containing 0.1 M of **4** in 10% v/v 1-octanol/*n*-dodecane. The two phases were contacted using a 1:1 ratio of organic/aqueous solution volume by end-over-end rotation in individual 1.8 mL capacity snap-top Eppendorf tubes using a rotating wheel in an airbox set at 25.5 ± 0.5 °C. Contacts were performed in triplicate with a contact time of 1 h. The samples were centrifuged at 1811*g* for 2 min at room temperature to separate the phases. Each triplicate was then subsampled using a 300 μL aliquot of the aqueous phase transferred to individual polypropylene tubes and diluted with 2% HNO_3_ for analysis.

## Supporting Information Available

Descriptor lists; experimental details (PDF)
Data sets and sources (XLSX)

## Notes

The data that support the findings of this study have been provided as the Supporting Information.
