# Supplementary material for: Advancing Rare-Earth Separation by Machine Learning
Source: JACS Au. 2022 Jun 15;2(6):1428–34. doi: 10.1021/jacsau.2c00122 (PMC9241157; doi:10.1021/jacsau.2c00122)
Supplement: Supplementary file 1 — au2c00122_si_001.pdf [file au2c00122_si_001.pdf]

Supporting Information

Advancing Rare Earth Separation by Machine Learning

Tongyu Liu,[1] Katherine R. Johnson,[2] Santa Jansone-Popova,[2] and De-en Jiang[1,*]

[1] Department of Chemistry, University of California, Riverside, California 92521, United States

[2] Chemical Sciences Division, Oak Ridge National Laboratory, Oak Ridge, Tennessee 37831, United States

*Email: djiang@ucr.edu

A. Data and Machine Learning Details

1. Descriptors of the extraction conditions

- Concentration of the ligand (mM)
- Volume ratios of organic solvents a and b
  - For the one-component organic phase, the volume ratio of organic solvent a is 10, the volume ratio of organic solvent b is 0, and all descriptors of organic solvent b are set as 0.
- Descriptors of organic solvent a:
  - Molar mass (g/mol)
  - Density (g/mL)
  - Boiling point (K)
  - Melting point (K)
  - Dipole moment (D)
  - Solubility in water (g/L)
  - log P (partition coefficient in *n*-octanol/water at ambient temperature, $P=[a]_{n\text{-octanol}}/[a]_{water}$)
- Descriptors of organic solvent b:
  - Molar mass (g/mol)
  - Density (g/mL)
  - Boiling point (K)
  - Melting point (K)
  - Dipole moment (D)
  - Solubility in water (g/L)
  - log P (partition coefficient in *n*-octanol/water at ambient temperature, $P=[b]_{n\text{-octanol}}/[b]_{water}$)
- Dipole moment of the inorganic acid in the aqueous phase (D)
- Inorganic acid concentration (M)
  - For the extraction data which provides only the pH value of the aqueous phase, the inorganic acid concentration is determined by this pH value
- Temperature (K)
- Ln(III) concentration (mM)

2. Descriptors of the lanthanides

- Atomic number
- Outer shell electrons of the atom
- Melting point (K) of the metal
- Boiling point (K) of the metal
- Density (g/cm^3) of the metal
- First ionization energy (kJ/mol) of the atom
- Second ionization energy (kJ/mol) of the atom
- Third ionization energy (kJ/mol) of the atom
- Electron affinity (kJ/mol) of the atom
- Atomic radius (Å) of the atom
- Covalent radius (Å) of the atom
- Pauling electronegativity of the atom
- Ionic radius (Å) of Ln(III)
- Standard entropy (J·mol^{-1}·K^{-1}) of the metal at ambient conditions

Table S1. Evaluations of the different combinations of the hyperparameters based on their performance on the validation set. The optimal set of hyperparameters are in bold.

Activation function		ELU	ELU	ELU	ELU	ELU	ReLU	PReLU	ELU
Learning rate		0.00001	0.00001	0.00001	0.001	0.000001	0.00001	0.00001	0.00001
Weight decay		0.01	0.01	0.01	0.01	0.01	0.01	0.01	0.01
epochs		5,000	5,000	5,000	5,000	5,000	5,000	5,000	10,000
Neurons on hidden layers	1st	256	512	512	512	512	512	512	512
	2nd		32	128	128	128	128	128	128
	3rd			16	16	16	16	16	16
R^2 on the validation set		0.36	0.58	0.77	0.18	0.65	0.77	0.73	0.80
MAE on the validation set		0.68	0.66	0.41	0.85	0.52	0.41	0.43	0.37
RMSE on the validation set		1.09	0.88	0.66	1.17	0.81	0.65	0.71	0.61
Activation function		ReLU	PReLU	ELU	ReLU	**PReLU**	PReLU	PReLU	PReLU
Learning rate		0.00001	0.00001	0.00001	0.00001	**0.00001**	0.00001	0.00001	0.00001
Weight decay		0.01	0.01	0.01	0.01	**0.01**	0.01	0.01	0.01
epochs		10,000	10,000	15,000	15,000	**15,000**	15,000	15,000	15,000
Neurons on hidden layers	1st	512	512	512	512	**512**	512	256	512
	2nd	128	128	128	128	**128**	64	64	128
	3rd	16	16	16	16	**16**	16	8	32
R^2 on the validation set		0.84	0.81	0.84	0.82	**0.85**	0.83	0.77	0.80
MAE on the validation set		0.36	0.39	0.36	0.36	**0.34**	0.33	0.42	0.38
RMSE on the validation set		0.54	0.59	0.55	0.58	**0.53**	0.52	0.65	0.61

B. Experimental Details

1. General Information

Commercially available compounds were purchased from Aldrich Chemical Co., Acros Organics, Alfa Aesar or TCI America and were used without further purification.

Nuclear Magnetic Resonance (NMR) Spectroscopy. NMR 1D spectra were recorded on an Avance III-400 MHz NMR spectrometer (Bruker Company) equipped with a 5 mm BBI or BBO probe. For ^{1}H and ^{13}C NMR spectra, residual solvent peak was used as an internal reference ($CDCl_3$: 7.25 ppm for ^{1}H NMR).

2. Synthesis

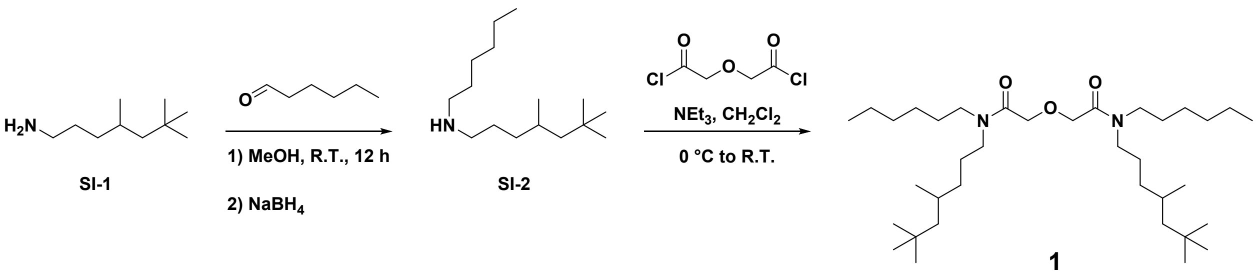

N-hexyl-4,6,6-trimethylheptan-1-amine: **SI-2**

To a mixture of hexanal (7.0 mL, 0.057 mol) in MeOH (100 mL) was added 4,6,6-trimethylheptan-1-amine (**SI-1**) (9.0 mL, 0.057 mol). The reaction mixture was stirred at room temperature for 12 hours. Afterwards, $NaBH_4$ (2.6 g, 0.069 mol) was added in small portions and reaction mixture stirred at room temperature for 2 hours. After the removal of reaction solvent on a rotary evaporator, 100 mL of Et_2O were added. The suspension was filtrated through a short silica gel-Celite® plug and washed with Et_2O (2x). The filtrate was concentrated under reduced pressure to yield product which was used in the next step without further purification.

2,2'-oxybis(N-hexyl-N-(4,6,6-trimethylheptyl)acetamide): **1**

To a solution of N-hexyl-4,6,6-trimethylheptan-1-amine **SI-2** (10.6 g, 0.05 mol, 2.1 equiv.) in anhydrous CH_2Cl_2 (0.3 M) was added Et_3N (2.1 equiv.). The reaction mixture was cooled in an ice-water bath prior to slow addition of 2,2-oxydiacetyl chloride (1 equiv.), then stirred at room temperature for 2 hours. The solvent was evaporated under reduced pressure. To the crude material, Et_2O (~0.3 M) was added, the precipitate was removed by filtration through a short Celite® plug and rinsed with Et_2O (2x). The filtrate was concentrated under reduced pressure and purified on CombiFlash® R_f automated flash chromatography system using normal phase silica gel as a stationary phase and gradient 10-60% EtOAc in hexanes as an eluent system (R_f = 0.6, 60% EtOAc/ Hex) to yield transparent oil (**1**, 4.3 g, 30%). Compound **1**: **^{1}H NMR** (400 MHz, $CDCl_3$) 4.30 (s, 4H), 3.39-3.03 (m, 8H), 1.60-1.44 (m, 8H), 1.37-1.17 (m, 22H), 1.17-1.04 (m, 6H), 0.95-0.75 (m, 24H). **^{13}C NMR** (100.67 MHz, $CDCl_3$) δ 168.6, 69.2, 51.3, 51.2, 47.3, 47.1, 46.2, 45.9, 36.8, 36.5, 31.74, 31.67, 31.2, 30.2, 29.2, 29.1, 27.7, 26.8, 26.7, 26.6, 25.4, 22.7, 14.2, 14.1.

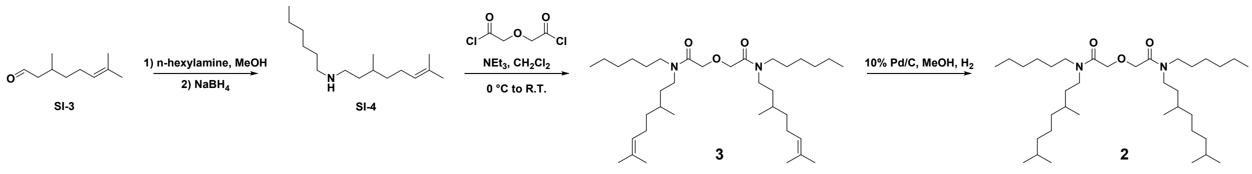

N-hexyl-3,7-dimethyloct-6-en-1-amine: **SI-4**

To a mixture of Citronellal **SI-3** (90 mL, 0.5 mol) in MeOH (950 mL) was added n-hexylamine (66 mL, 0.5 mol). The reaction mixture was stirred at room temperature for 12 hours. Afterwards, $NaBH_4$ (22.7 g, 0.6 mol) was added in small portions and reaction mixture stirred at room temperature for 2 hours. After the removal of reaction solvent on a rotary evaporator, 400 mL of Et_2O were added. The suspension was filtrated through a short silica gel-Celite® plug and washed with Et_2O (2x). The filtrate was concentrated under reduced pressure to yield product which was used in the next step without further purification.

2,2'-oxybis(N-(3,7-dimethyloct-6-en-1-yl)-N-hexylacetamide): **3**

To a solution of *N*-hexyl-3,7-dimethyloct-6-en-1-amine **SI-4** (103 g, 0.43 mol, 2.1 equiv.) in anhydrous CH_2Cl_2 (0.3 M) was added Et_3N (2.1 equiv.). The reaction mixture was cooled in an ice-water bath prior to slow addition of 2,2-oxydiacetyl chloride (1 equiv.), then stirred at room temperature for 2 hours. The solvent was evaporated under reduced pressure. To the crude material, Et_2O (~0.3 M) was added, the precipitate was removed by filtration through a short Celite® plug and rinsed with Et_2O (2x). The filtrate was concentrated under reduced pressure and purified on CombiFlash® R_f automated flash chromatography system using normal phase silica gel as a stationary phase and gradient 10-60% EtOAc in hexanes as an eluent system (R_f = 0.6, 60% EtOAc/ Hex) to yield transparent oil (**3**, 67.3 g, 60%). Compound **3**: **^{1}H NMR** (400 MHz, $CDCl_3$) 4.30 (s, 4H), 3.39-3.03 (m, 8H), 1.60-1.44 (m, 8H), 1.37-1.17 (m, 22H), 1.17-1.04 (m, 6H), 0.95-0.75 (m, 24H). **^{13}C NMR** (100.67 MHz, $CDCl_3$) δ 168.7, 131.7, 131.4, 124.8, 124.5, 69.23, 69.15, 47.0, 46.0, 45.4, 44.2, 37.1, 37.0, 36.1, 34.6, 31.7, 31.6, 30.8, 30.6, 29.0, 27.7, 26.8, 26.6, 25.9, 25.5, 22.7, 22.7, 19.6, 17.8, 14.2, 14.1.

2,2'-oxybis(N-(3,7-dimethyloctyl)-N-hexylacetamide): **2**

Compound **2** was synthesized via hydrogenation of *2,2'-oxybis(N-(3,7-dimethyloct-6-en-1-yl)-N-hexylacetamide)* **3** using 10% Pd/C (2.4 g/0.1 mol of SI-7) in MeOH (0.2 M) and H_2 (1 atm, balloon). The reaction mixture was filtrated through a short Celite® plug and washed with MeOH. The filtrate was concentrated under reduced pressure to yield product as light-yellow oil (99% yield). Compound **2**: **^{1}H NMR** (400 MHz, $CDCl_3$) 4.30 (s, 4H), 3.39-3.03 (m, 8H), 1.60-1.44 (m, 8H), 1.37-1.17 (m, 22H), 1.17-1.04 (m, 6H), 0.95-0.75 (m, 24H). **^{13}C NMR** (100.67 MHz, $CDCl_3$) δ 169.0, 69.3, 69.2, 47.1, 46.2, 45.5, 44.4, 39.4, 39.3, 37.3, 37.2, 36.1, 34.6, 31.7, 31.6, 31.2, 31.1, 29.0, 28.1, 27.7, 26.8, 26.6, 24.8, 22.8, 22.7, 19.7, 14.2, 14.1.

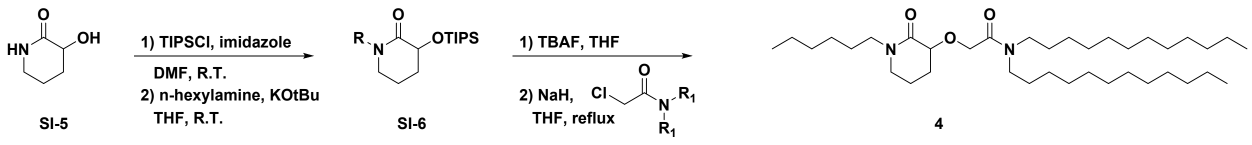

1-hexyl-3-((triisopropylsilyl)oxy)piperidin-2-one: **SI-6**

3-hydroxypiperidin-2-one **SI-5** (2.5 g, 0.02 mol) and imidazole (1.05 equiv) were dissolved in anhydrous DMF (0.2 M). To this solution was then added TIPS-Cl (1.05 equiv) and the reaction mixture was stirred at room temperature for 12 hours. To the reaction mixture was added water and product was extracted with E_2O (3x). The combined organic phase was washed with brine, dried over $MgSO_4$, filtered, and solvent removed under reduced pressure. The product was used in the next step without further purification. To the ice-cold solution of the TIPS protected product (5.9 g, 0.02 mol) in anhydrous THF (0.2 M) was added tBuOK (2.68 g, 0.02 mol). The reaction mixture was stirred for 30 min before the addition of n-hexyl iodide. Afterwards, the reaction mixture was stirred for 12 hours at room temperature. To the reaction mixture was added water and product was extracted with E_2O (3x). The combined organic phase was washed with brine, dried over $MgSO_4$, filtered, and solvent removed under reduced pressure. The product (**SI-6**) was used in the next step without further purification.

N,N-didodecyl-2-((1-hexyl-2-oxopiperidin-3-yl)oxy)acetamide: **4**

To 1-hexyl-3-((triisopropylsilyl)oxy)piperidin-2-one **SI-6** (0.02 mol) dissolved in anhydrous THF (0.4 M) was added TBAF (1 M in THF, 1.2 equiv). The reaction mixture was stirred at room temperature for 12 hours. Afterwards, the solvent was removed under reduced pressure and product was purified on CombiFlash® R_f automated flash chromatography system using normal phase silica gel as a stationary phase and gradient 0-80% EtOAc in hexanes as an eluent system to yield light yellow oil (3.9 g, 90%). Next, the round bottom flask was charged with NaH (0.25 g, 6.3 mmol) and anhydrous THF (0.2 M) under inert atmosphere. To the reaction mixture was then added dropwise the above obtained product (1.26 g, 6.3 mmol) dissolved in 5 mL of anhydrous THF. The reaction mixture was stirred at room temperature for 30 minutes. Afterwards, 2-chloro-N,N-didodecylacetamide **SI-7** (3.0 g, 6.3 mmol) dissolved in anhydrous THF (5 mL) was added to the reaction mixture. The reaction mixture was heated at 35 ˚C for 12 hours. To the reaction mixture was added water and product was extracted with E_2O (3x). The combined organic phase was washed with brine, dried over $MgSO_4$, filtered, and solvent removed under reduced pressure. and product was purified on CombiFlash® R_f automated flash chromatography system using normal phase silica gel as a stationary phase and gradient 0-80% EtOAc in hexanes as an eluent system to yield light yellow oil (2.7 g, 73%). **^{1}H NMR** (400 MHz, $CDCl_3$) 4.73-4.55 (m, 2H), 4.00-3.92 (m, 1H), 3.38-3.05 (m, 8H), 2.24-2.12 (m, 1H), 2.07-1.94 (m, 2H), 1.86-1.65 (m, 3H), 1.60-1.45 (m, 6H), 1.35-1.18 (m, 40H), 0.93-0.83 (m, 9H). **^{13}C NMR** (100.67 MHz, $CDCl_3$) δ 170.2, 169.8, 75.4, 69.3, 47.8, 47.5, 47.1, 46.3, 32.1, 31.7, 29.8, 29.7, 29.7, 29.6, 29.5, 28.9, 28.4, 27.7, 27.2, 27.03, 27.01, 26.7, 22.8, 22.7, 19.8, 14.3, 14.2.

3. Solvent Extraction

All reagents were of analytical grade and used without further purification.

*Procedure for extraction of Ln(III) with **1 - 3***

A 750 microliter (μL) aqueous phase containing 7 mM Ln(III) (0.5 mM of each Ln(III)) in 3 M HCl was contacted with an equal volume of preequilibrated organic phase containing 0.1 M of the desired DGA (**1** – **3**) in 30% v/v Exxal 13/Isopar L. The two phases were contacted using a 1:1 ratio of organic/aqueous solution volume by end-over-end rotation in individual 1.8 mL capacity snap-top Eppendorf tubes using a rotating wheel in an airbox set at 25.5 ± 0.5°C. Contacts were performed in triplicate with a contact time of 1 hour. The samples were centrifuged at 1,811 × *g* for two minutes at room temperature to separate the phases. Each triplicate was then subsampled using a 500 μL aliquot of the aqueous phase transferred to individual polypropylene tubes and diluted with 4% HNO_3 for analysis. Two samples of the initial lanthanide solution were similarly prepared. The area under each observed emission peak was used for determining the concentration of Ln(III) in each solution.

*Procedure for extraction with Ln(III) with **4***

A 500 microliter (μL) aqueous phase containing 7 mM Ln(III) (0.5 mM of each Ln(III)) in 1 M HNO_3 was contacted with an equal volume of preequilibrated organic phase containing 0.1 M of **4** in 10% v/v 1-octanol/n-dodecane. The two phases were contacted using a 1:1 ratio of organic/aqueous solution volume by end-over-end rotation in individual 1.8 mL capacity snap-top Eppendorf tubes using a rotating wheel in an airbox set at 25.5 ± 0.5°C. Contacts were performed in triplicate with a contact time of 1 hour. The samples were centrifuged at 1,811 × *g* for two minutes at room temperature to separate the phases. Each triplicate was then subsampled using a 300 μL aliquot of the aqueous phase transferred to individual polypropylene tubes and diluted with 2% HNO_3 for analysis. Two samples of the initial lanthanide solution were similarly prepared. The area under each observed emission peak was used for determining the concentration of Ln(III) in each solution.

Inductively Coupled Plasma Optical Emission Spectroscopy (ICP-OES)

Aqueous samples were analyzed on an iCAP 7400 spectrometer (ThermoFisher Scientific) equipped with a prepFAST M5X autodilution autosampler (Elemental Scientific). Calibration curves were collected using at least a 5 point curve using Rare Earth Element Mix (TraceCert, Sigma Aldrich) in either a 2% or 4% HNO_3 matrix with $R^2 > 0.95$ Each sample was measured in triplicate. The following wavelengths were used.

Lanthanide	Wavelength (nm)
La	412.323
Ce	404.076
Pr	417.939
Nd	406.109
Sm	330.639
Eu	381.967
Gd	342.247
Tb	350.917
Dy	353.170

Ho	345.600
Er	337.271
Tm	313.126
Yb	297.056
Lu	261.542

The distribution ratio (D) was measured by measuring the concentration of the metal ion in the aqueous phase after extraction and by comparing it to the initial concentration. As such, D values were determined using the following equation:

$$D = \frac{C_i - C_f}{C_f},$$

where C_i and C_f are the concentrations of the metal ions in the aqueous phase before and after extraction, respectively.

1. NMR spectra

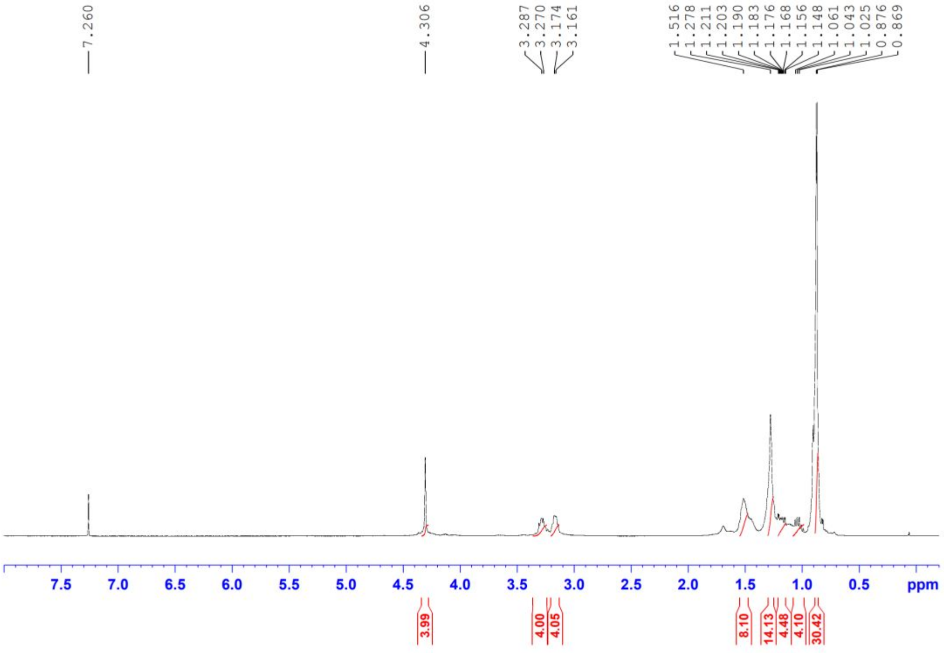

Figure S1. ^{1}H NMR spectrum of **1** in $CDCl_3$.

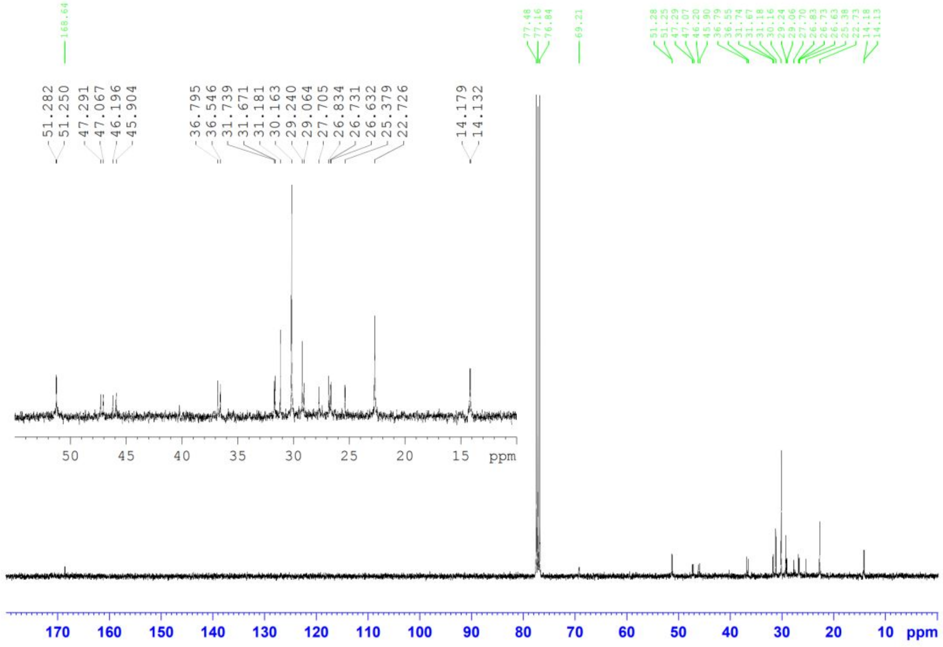

Figure S2. ^{13}C NMR spectrum of **1** in $CDCl_3$.

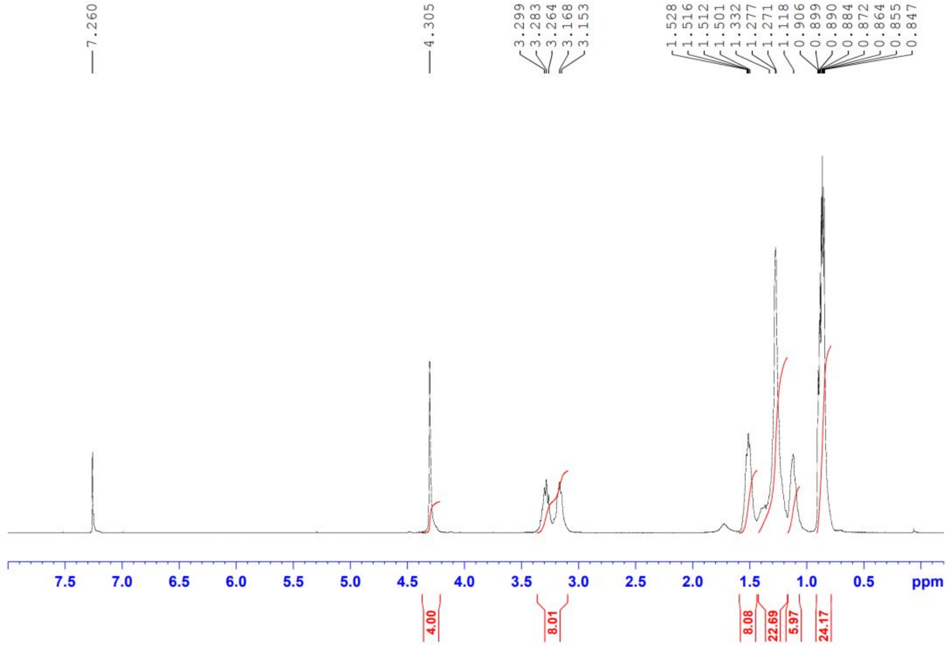

Figure S3. ^{1}H NMR spectrum of **2** in $CDCl_3$.

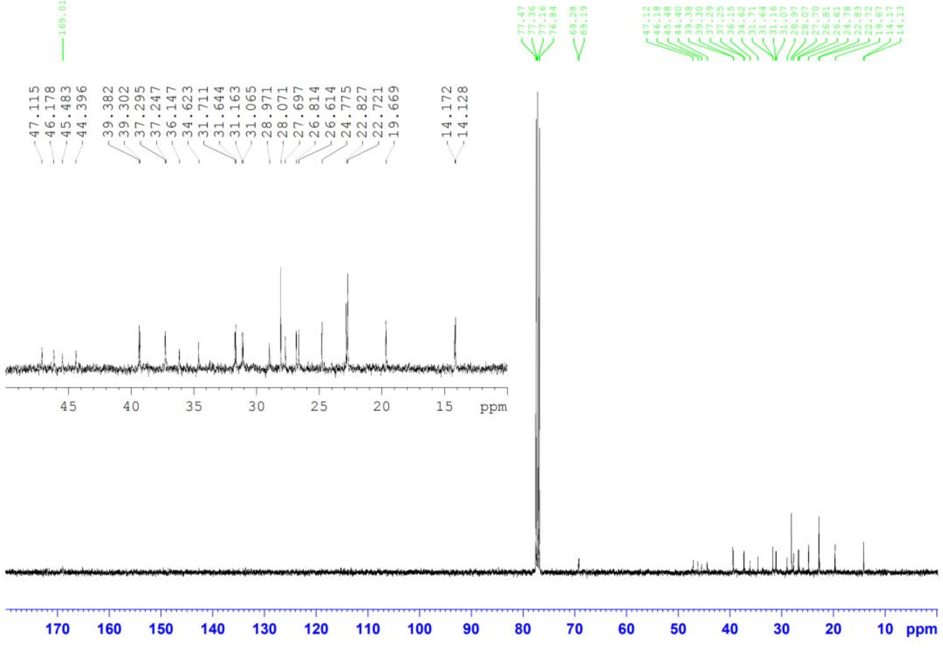

Figure S4. ^{13}C NMR spectrum of **2** in $CDCl_3$.

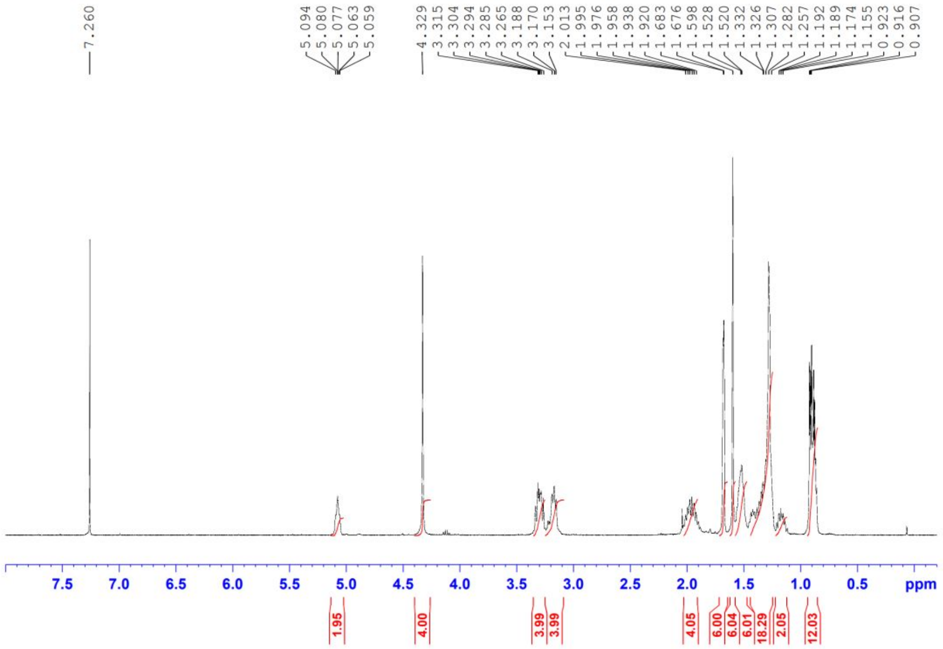

Figure S5. ^{1}H NMR spectrum of **3** in $CDCl_3$.

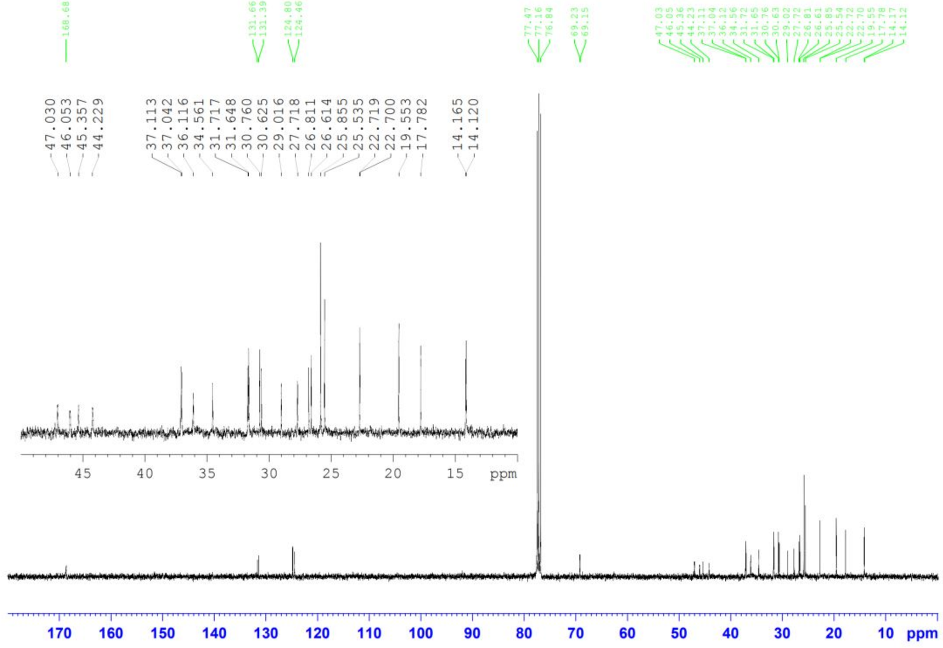

Figure S6. ^{13}C NMR spectrum of **3** in $CDCl_3$.

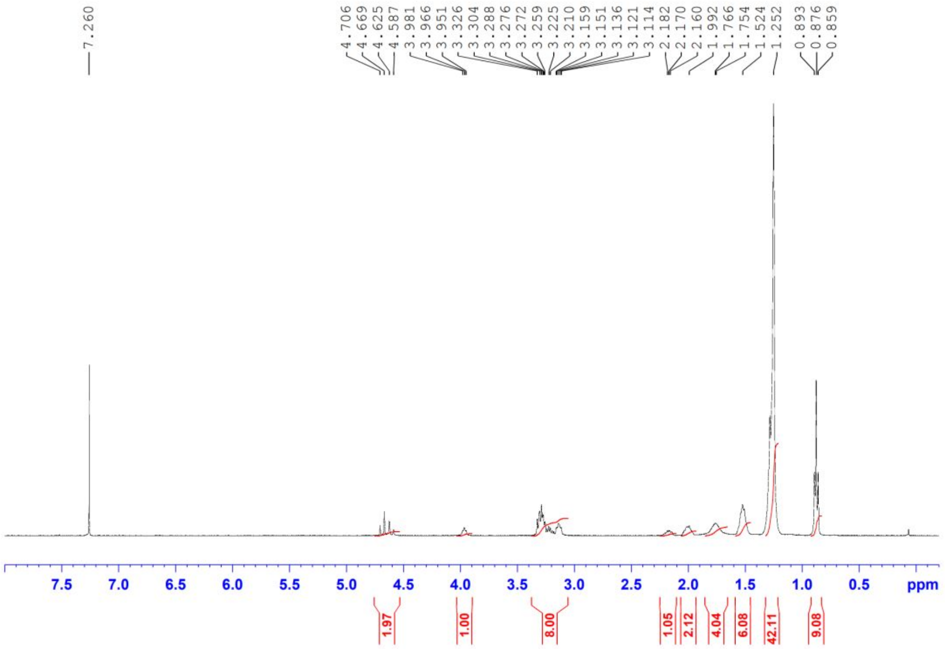

Figure S7. ^{1}H NMR spectrum of **4** in $CDCl_3$.

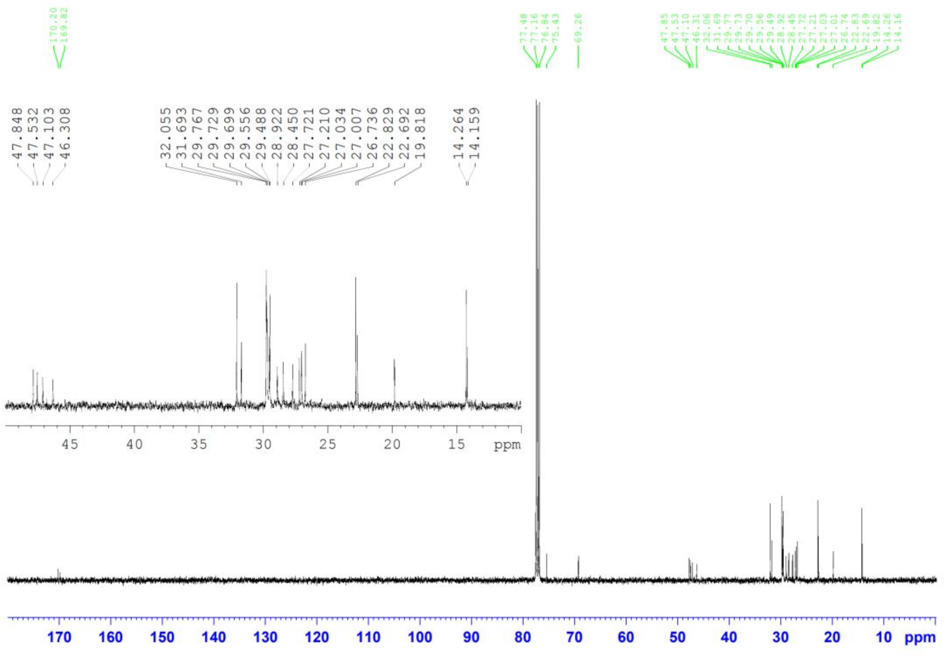

Figure S8. ^{13}C NMR spectrum of **4** in $CDCl_3$.
